# Ex vivo expansion of circulating tumour cells (CTCs)

**DOI:** 10.1038/s41598-023-30733-6

**Published:** 2023-03-06

**Authors:** Bashir M. Mohamed, Mark P. Ward, Mark Bates, Cathy D. Spillane, Tanya Kelly, Cara Martin, Michael Gallagher, Sheena Heffernan, Lucy Norris, John Kennedy, Feras Abu Saadeh, Noreen Gleeson, Doug A. Brooks, Robert D. Brooks, Stavros Selemidis, Sharon O’Toole, John J. O’Leary

**Affiliations:** 1 Department of Histopathology, Trinity College Dublin, Emer Casey Molecular Pathology Research Laboratory, Coombe Women & Infants University Hospital, Dublin, Ireland; 2 Trinity St James’s Cancer Institute, Dublin 8, Ireland; 3 Department of Obstetrics and Gynaecology, Trinity College Dublin, Dublin, Ireland; 4 HOPE Directorate, St. James’s Hospital, Dublin 8, Ireland; 5 Division of Gynaecological Oncology, St. James’s Hospital, Dublin 8, Ireland; 6 Clinical and Health Sciences, University of South Australia, Adelaide, SA 5001 Australia; 7 School of Health and Biomedical Sciences, RMIT University, Bundoora, VIC 3083 Australia

**Keywords:** Biological techniques, Cancer

## Abstract

Circulating tumour cells (CTCs) are a critical intermediate step in the process of cancer metastasis. The reliability of CTC isolation/purification has limited both the potential to report on metastatic progression and the development of CTCs as targets for therapeutic intervention. Here we report a new methodology, which optimises the culture conditions for CTCs using primary cancer cells as a model system. We exploited the known biology that CTCs thrive in hypoxic conditions, with their survival and proliferation being reliant on the activation of hypoxia-inducible factor 1 alpha (HIF-1α). We isolated epithelial-like and quasi-mesenchymal CTC phenotypes from the blood of a cancer patient and successfully cultured these cells for more than 8 weeks. The presence of CTC clusters was required to establish and maintain long-term cultures. This novel methodology for the long-term culture of CTCs will aid in the development of downstream applications, including CTC theranostics.

## Introduction

Metastasis is integrally linked to cancer-related deaths^1^, making the detection and treatment of advanced metastatic disease a major global challenge. Currently, the mechanisms underlying metastatic events are poorly understood, but circulating tumour cells (CTCs) in the blood are believed to be responsible for cancer dissemination from the primary tumour to a distant site, for the seeding of secondary cancers^2^. CTCs in the blood of cancer patients appear to exhibit dynamic changes in morphology and interact with immune cells, platelets and erythrocytes as they transit from the primary tumour to form secondary metastatic cancers^3^. In addition to heterotypic cellular interactions, CTCs are also able to self-aggregate to form homotypic/heterotypic clusters; also called circulating tumour microemboli^4,5^. The size and number of CTC clusters directly correlates with the development of metastasis^4,5^ and is indicative of advanced cancer progression. CTC clusters have a survival advantage in the circulation and are effectively a measure of cancer cell adaptation to an altered microenvironment in the blood, where they encounter low nutrient availability, reduced oxygen levels and tension from circulatory shear forces^3,6^. The isolation and real-time monitoring of CTCs in cancer patients will improve early cancer detection, the monitoring of disease progression and therapeutic target selection, and will also enable real-time treatment response prediction and assessment of therapeutic efficacy. CTC enumeration is now considered to be a key prognostic factor in breast, colorectal, prostate and lung cancers^7,8^. Several CTC isolation methods have been developed, including; Immunomagnetic capture methods (CellSearch, Adna test, and Isoflux system), Microfluidic enrichment technologies (chip-based technologies, ClearCell FX and Parsortix device), Chip Based Isolation Technologies (Herringbone microfluidic chip, Cluster Chip, and CTC iChip), Microfiltration and Size Based Isolation Technologies (ISET and ScreenCell) and RosetteSep (negative enrichment technique)^9–25^.

CTCs are relatively rare in most cancer patients^26^, with typically only one to ten CTCs being isolated from 7.5 mL of whole blood sample (equating to 1–100 CTCs per 10^9^ white blood cells). Only a few laboratories have successfully cultured viable CTCs to produce large populations of cancer cells for ex vivo expansion^27–32^. There is an urgent unmet need for a reliable method that enables ex vivo expansion and stable long-term culture of CTCs, to improve developmental research and clinical service provision.

There is a lack of standardised CTC culture conditions for different cancer cell types and optimal conditions remain to be defined, particularly for long-term CTC expansion cultures. Similar to short-term maintenance cultures, a cocktail of growth factors appears to be necessary in order to stably expand CTCs for long-term culture. Essential cytokines, hormones, tissue organ extracts, growth factors (e.g. epidermal growth factor (EGF), fibroblast growth factors (FGF2 and FGF10), granulocyte–macrophage colony-stimulating factor (GM-CSF), insulin/insulin-like growth factor 1 (IGF-1) are thought to play important roles for the survival and growth of CTCs in vitro^26–28^. Low-adherent culture conditions also appear to be important for long-term ex vivo culture of CTCs, because CTCs undergo senescence after a few passages in adherent monolayer cultures^27^. Hypoxic conditions also play an important role in long-term CTC culture^26,27^ and hypoxia-inducible factor-1α (HIF-1α) has been identified as a key regulator of this process, activating a spectrum of downstream genes to promote cell proliferation, angiogenesis and metastasis^33–37^. These factors need to be optimised to enable the development of an effective and reliable CTC culture method, which ultimately needs to be assessed for CTCs from different cancers.

In this study, we have focused on hypoxia as a critical factor in CTC biology, and have developed a Cobalt(II) chloride (CoCl_2_) modified culture system, using an approved CTC isolation device the ScreenCell LCD kit^38^ and low adherence culture plates for the long-term ex vivo culture and expansion of CTCs. CoCl_2_ is an alternative to technically difficult hypoxia culture systems, as it is an inexpensive chemical agent, which maintains steady oxygen tension, allowing real-time monitoring of cells. CoCl_2_ has been widely used as a chemical hypoxia-mimicking agent, to create a “hypoxia-like” state in vitro by stabilizing HIF-1α, which is the master regulator of the cellular adaptive response to hypoxia^39^. Importantly, mononuclear cells did not survive in a hypoxic condition, and even when viable remain adherent to the filter^40,41^. This CoCl_2_ protocol helps to overcome some of the difficulties involved in culturing and analysing these relatively rare cancer cells^42^.

## Results

### Optimisation of cell culture conditions using a primary ovarian cell model

#### CoCl_2_ induced HIF-1α expression

**Figure 1:**
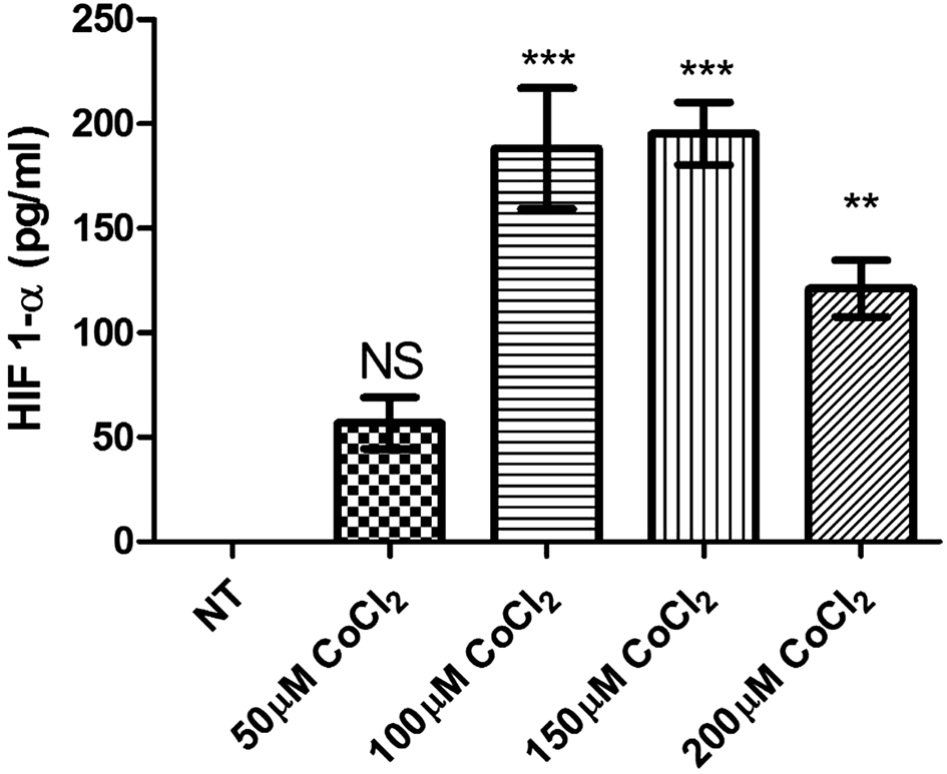
Effect of CoCl_2_ on HIF-1α protein abundance. Primary ovarian cancer cells were incubated with different concentrations of CoCl_2_ (50,100, 150 and 200 µM) for 48 h. Cell lysates were collected and HIF‐1α protein was determined by ELISA. Data is presented as mean ± SEM (n = 3) and was analysed using one‐way ANOVA with Tukey’s post-hoc test, with respect to the corresponding not treated controls (NT), and statistically significant data is reported by *** for *p* < 0.001, ** for *p* < 0.01 and NS for not significant.

In order to interrogate the effects of CoCl_2_ exposure on HIF-1α protein levels, primary ovarian cancer cells were exposed to different concentrations of CoCl_2_ (50, 100, 150 and 200 µM) for 48 h^39^. CoCl_2_ exposure induced a significant increase in HIF-1α protein expression at 100–200 µM (Fig. 1).

#### Cell viability and cell cycle progression were maintained upon exposure to CoCl_2_

**Figure 2:**
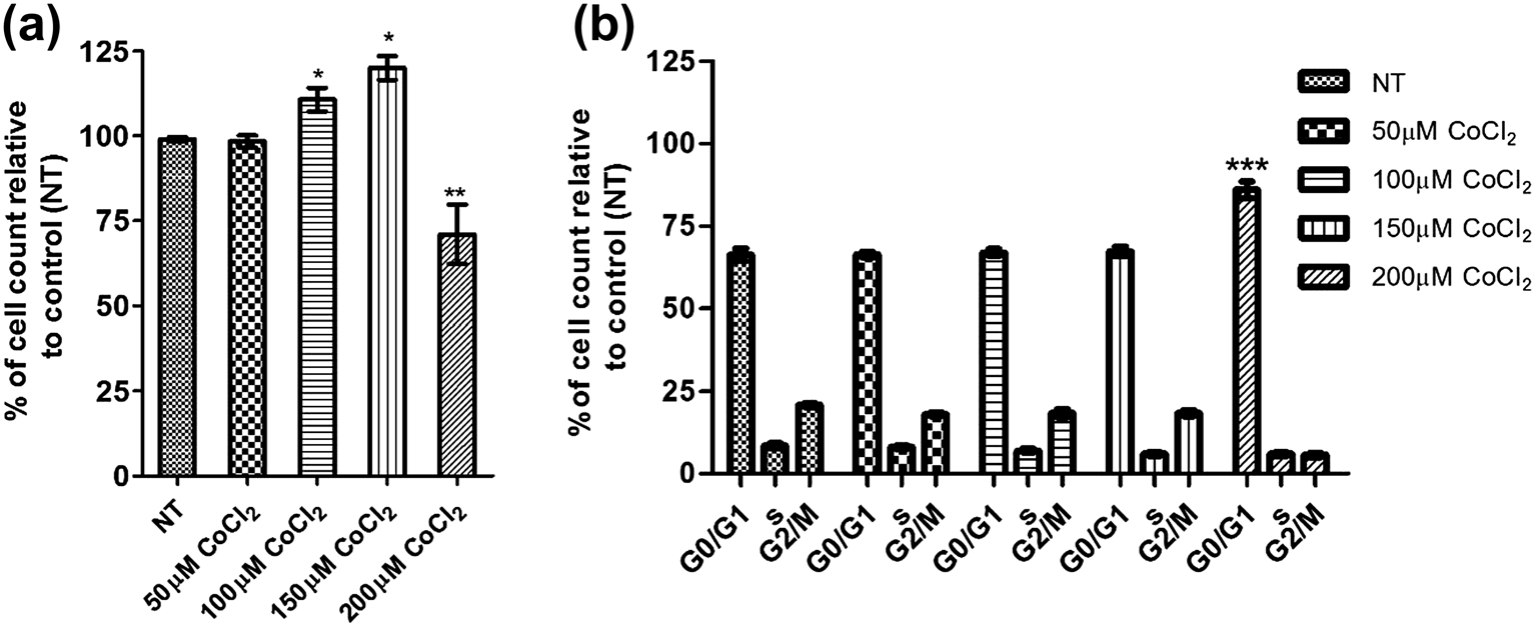
Effect of CoCl_2_ on cell viability and cell cycle. Primary ovarian cancer cells were either not treated (NT) or treated with different concentrations of CoCl_2_ (50,100,150 and 200 µM) for 48 h. Cells were stained with Hoechst 33342 and scanned and analysed using the Cytell imaging system and Cell Cycle BioApp software. (**a**) Viable cells were automatically counted and data were presented as mean ± SEM (n = 3) and were analysed using one‐way ANOVA with Tukey’s post-hoc test, “*”*p* < 0.05. (**b**) Cell cycle distribution was analysed using the cell cycle Bioapplication. Data were presented as mean ± SEM (n = 3). 2-way ANOVA with Bonferroni post-test analysis was carried out on the experimental data, with respect to the corresponding controls, and statistically, significant data is reported by *for *p* < 0.05; ** for *p* < 0.01 and *** for *p* < 0.001.

In primary ovarian cells treated with 100 and 150 µM CoCl_2,_ there was a significant (p < 0.001) increase in cell count, demonstrating sustainable proliferation activity, whereas a significant (p < 0.05) decrease was observed at a higher concentration of 200 µM CoCl_2_ in comparison to the untreated control cells (Fig. 2a). At the higher 200 µM concentration of CoCl_2_ there was a significant cell cycle arrest at G0/G1 (p < 0.001), indicating altered cell viability (Fig. 2b).

#### γ-H2AX expression

**Figure 3:**
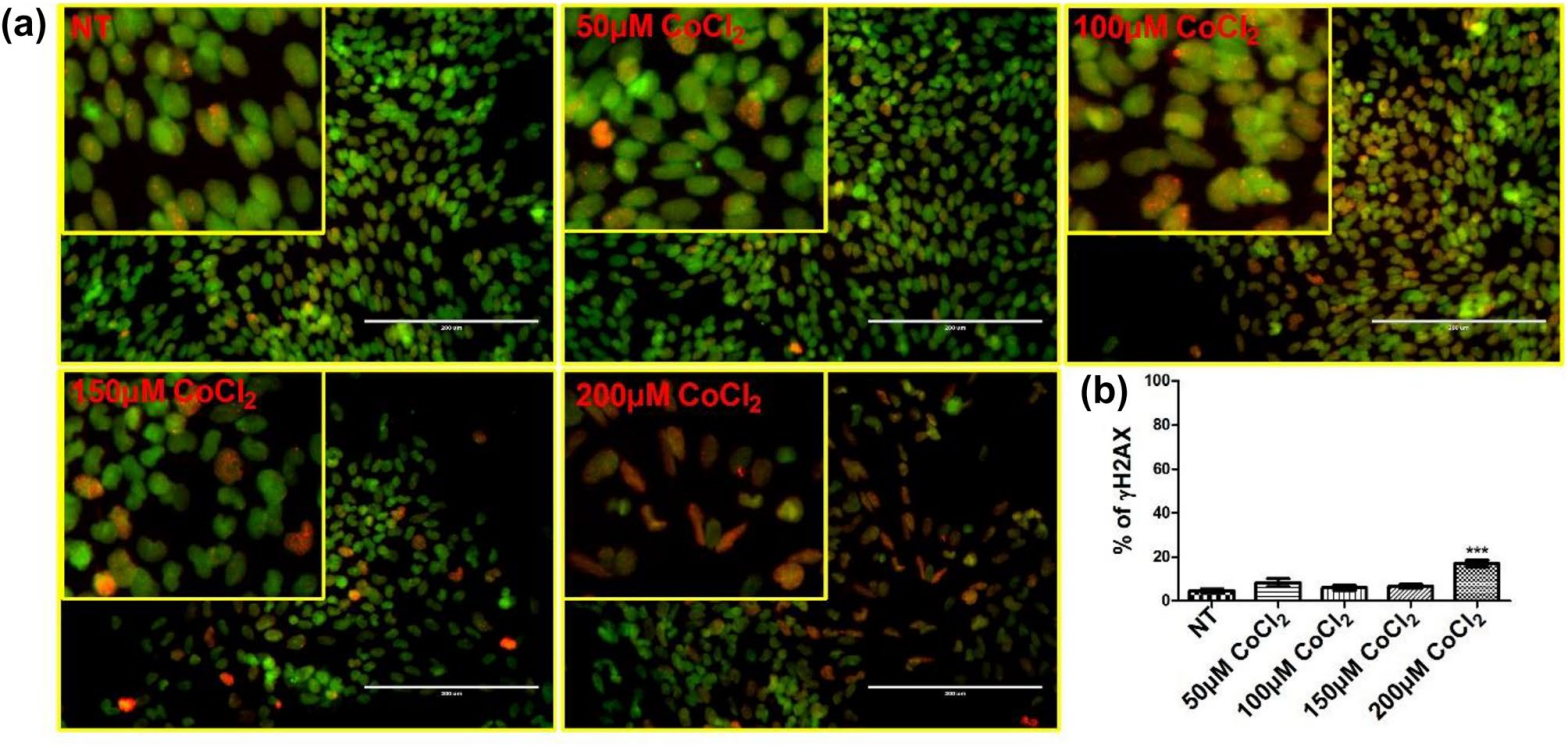
Gamma‐H2AX immunofluorescence staining. Primary ovarian cancer cells were incubated with various concentrations of CoCl_2_ (50,100,150 and 200 µM) for 48 h. (**a**) Cells were stained with Hoechst 33342 (Green) and γ‐H2AX (red) and then five microscopic fields per well were scanned and analysed using the Cytell imaging system. (**b**) The number of γ‐H2AX stained cells were automatically counted and data presented as mean ± SEM (n = 3). Data was analysed using one‐way ANOVA with Tukey’s post-test analysis relative to corresponding not treated controls (NT), ***p < 0.001.

Gamma H2AX (γ-H2AX) is a potential regulator of DNA repair and is a useful tool for detecting DNA damage^43^. To evaluate if CoCl_2_ induced DNA damage, we exposed primary ovarian cancer cells to a range of CoCl_2_ concentrations (50, 100, 150 and 200 µM) for 48 h and examined the expression of γ-H2AX. We did not detect any induction of DNA damage by CoCl_2_ at or less than 150 µM, while significant DNA damage was observed when the cells were exposed to 200 µM of CoCl_2_ (Fig. 3a,b).

#### PHH3 expression upon CoCl_2_ exposure

PHH3 is a known marker of cellular proliferation and was used to assess the proliferation activity under hypoxic conditions^44^. The expression of PHH3 was examined in primary ovarian cancer cells after exposure to different concentrations of CoCl_2_. The expression of PHH3 was not altered at 50 µM of CoCl_2_, while it was significantly increased at 100 and 150 µM of CoCl_2_ (p < 0.05) as determined by immunofluorescence (Supplementary Fig. 1a,b) and western blotting (Supplementary Fig. 2). When cells were exposed to 200 µM of CoCl_2_, there was a reduction in the amount of PHH3 expression (p < 0.001).

#### EpCAM, HER2 and vimentin expression in primary ovarian cells exposed to CoCl_2_

**Figure 4:**
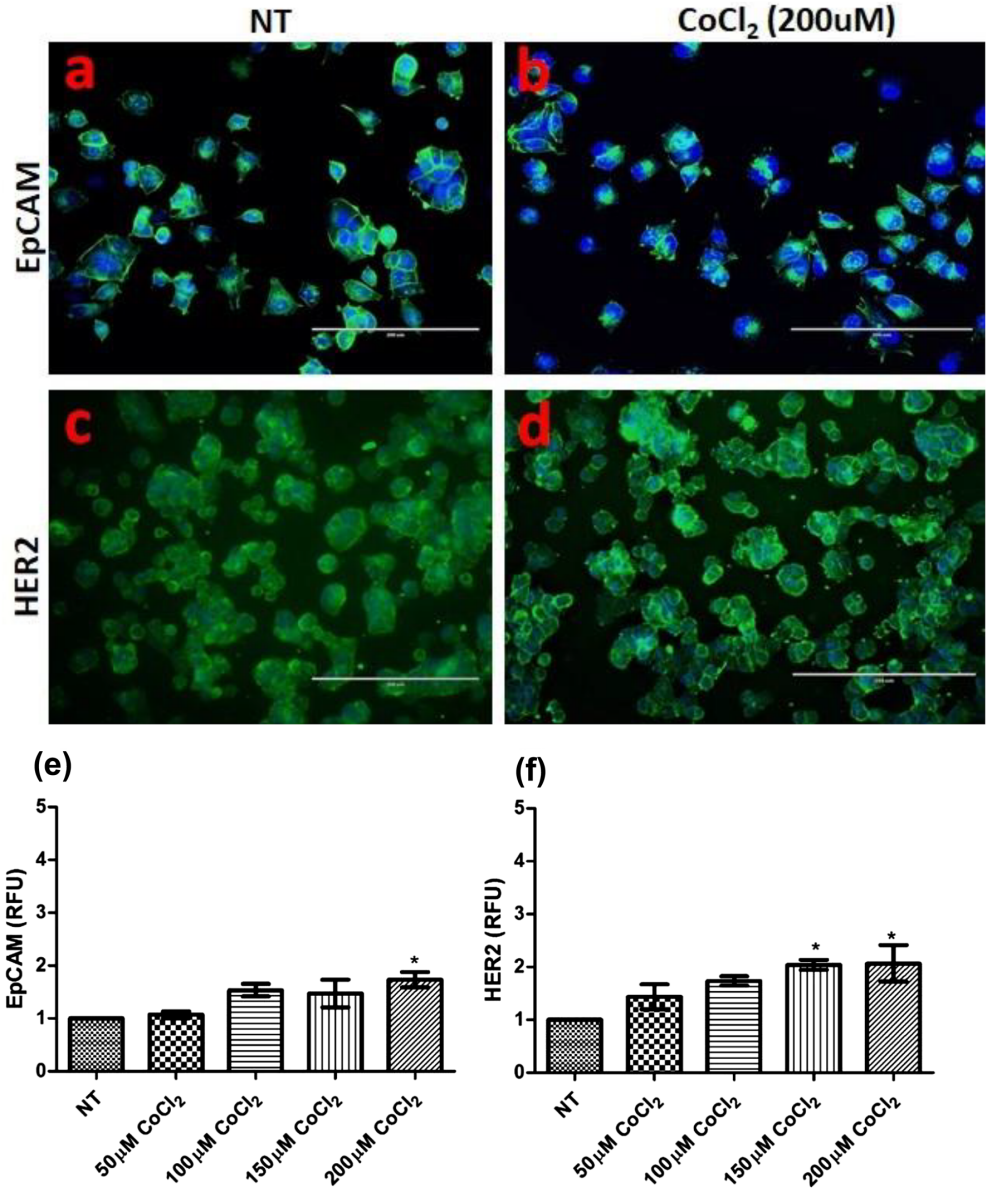
Influence of CoCl_2_ on EpCAM and HER2 expression in primary ovarian cancer cells. Cells were incubated with (50,100,150 and 200 µM) CoCl_2_ for 48 h. Cells were stained either with anti-EpCAM (**a**,**b**) or anti-HER2 (**c**,**d**). Five microscopic fields per well were scanned and analysed and the expression level was performed using the Cytell imaging system and IN Cell Investigator image analysis software (**e**,**f**). Data is presented as mean ± SEM (n = 3). One-way ANOVA with Tukey’s post-hoc test was carried out, with respect to the corresponding not treated controls (NT), and statistically significant data was reported by * for *p* < 0.05.

The relationship between CoCl_2_ exposure (HIF-1α inducer) and the expression of vimentin (Supplementary Fig. 1a), epithelial cell adhesion molecule (EpCAM) (Fig. 4a,d), and the growth factor receptor (HER2) (Fig. 4b,c) was examined in ovarian cancer cells. There were no detectable changes in vimentin expression observed for any of the CoCl_2_ concentrations examined. EpCAM expression was not changed at concentrations up to 150 µM, whereas EpCAM expression was significantly increased (p < 0.05) at 200 µM CoCl_2_ (Fig. 4e). HER2 expression was significantly increased (p < 0.05) at concentrations at or above 150 µM (Fig. 4f). Based on the observations above from primary ovarian cells, 100 µM of CoCl_2_ had the most optimal effect on HIF-1α, cell proliferation, cell viability and the cancer cell biomarker expression, and was chosen for the ex vivo examination of ovarian and breast cancer patient CTCs.

#### Ex-vivo culture of CTCs isolated from ovarian and breast cancer patient blood samples

When assessed morphologically, only three of 13 (27%) ovarian cancer patient blood samples had evidence of CTCs, with a median count of 15 (range 8–28). Our aim was to isolate CTCs using the ScreenCell system and to achieve a long-term CTC culture using hypoxia-modified cell culture conditions. On morphological assessment, seven of 18 (39%) breast cancer patient blood samples had evidence of CTCs, with a median cell count of 4 (range, 3–13). Only one of these seven breast cancer patient blood samples (14%) had a mixture of potential singlet and cluster CTCs. CTCs were incubated in culture medium supplemented with 100 µM of CoCl_2_, as a chemical inducer of HIF-1α, and cultured in low adherence plates.

**Figure 5:**
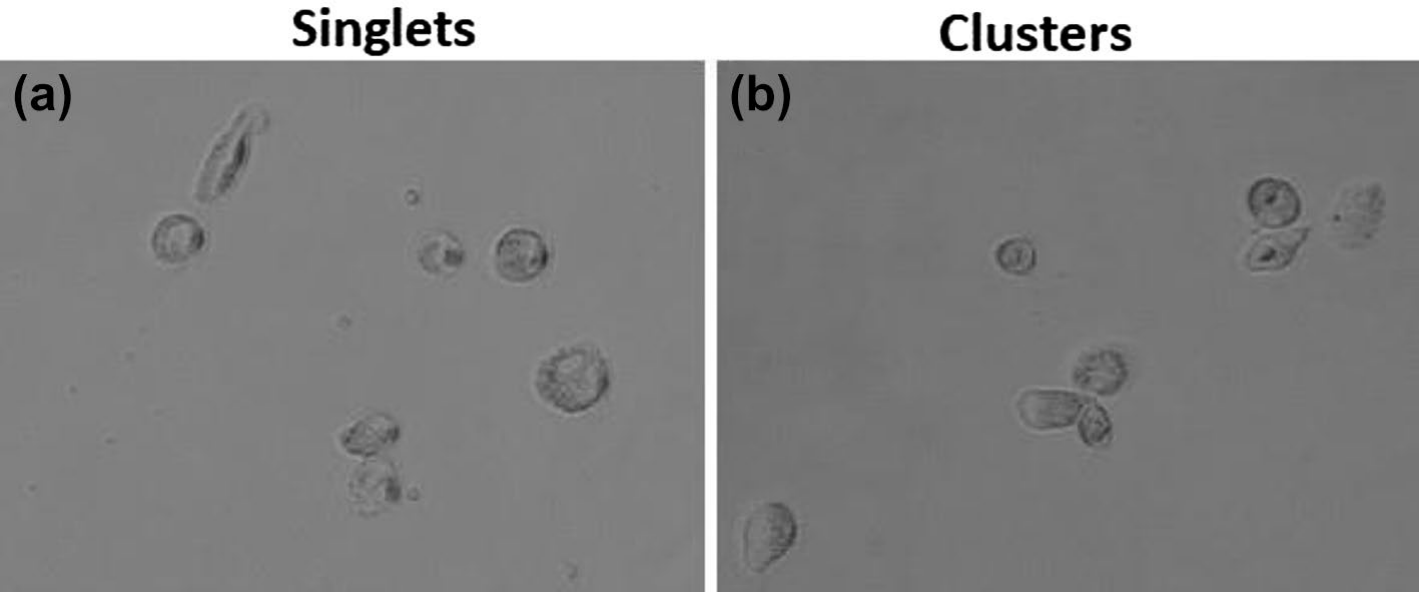
Brightfield images of isolated CTCs. (**a**) CTC singlets and (**b**) CTC clusters were captured and isolated from a breast cancer patient using the ScreenCell device. Images were taken with an inverted microscope (20×).

**Figure 6:**
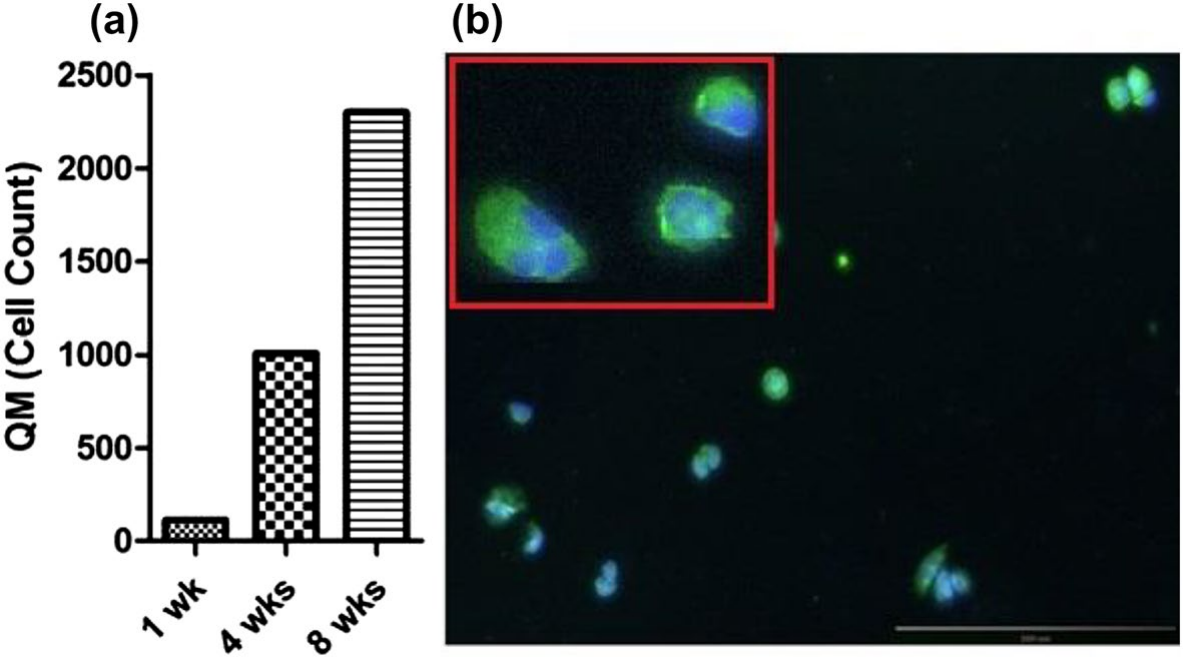
Quasi-mesenchymal (QM) cells derived from a breast cancer patient blood sample. (**a**) The figure illustrates the number of QM cells cultured over time (1wk, 4 wks and 8 wks) as measured using a haemocytomer. (**b**) Immunostaining revealed that these cells express HER2 at 8 weeks. Cells were counterstained with Hoechst 33342, blue for visualisation of cell nuclei. Imaging was performed using an inverted microscope (10×).

Using these culture conditions, two molecular subtypes were observed from the CTC cultures derived from one of the breast cancer patients (hormone receptor-negative and HER2 positive); classical epithelial-like singlet and cluster CTCs (Fig. 5) and quasi-mesenchymal CTCs (Fig. 6). For the CTCs cultured from a blood sample from one of the breast cancer patients, we observed quasi-mesenchymal cells 1-week post culturing and over 100 cells were present. At 4 weeks post isolation, the cell number exceeded one thousand and at 8 weeks post isolation exceeded 2000 cells (Fig. 5a), suggesting that the cells were actively proliferating. Fluorescent imaging revealed that these cells expressed HER2 (Fig. 6b).

**Figure 7:**
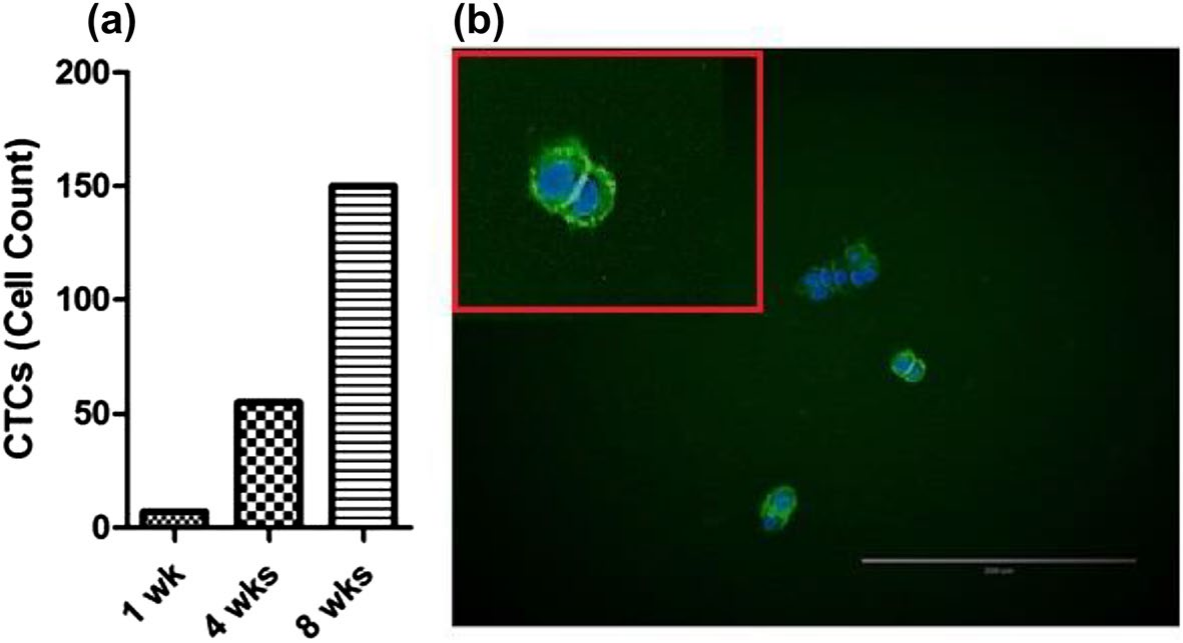
Characterisation of Epithelial-Like CTCs derived from the blood of a breast cancer patient. The figure illustrates the number of CTCs grown over time (1 wk, 4 wks and 8 wks) in culture as measured by hemocytometry. (**b**) Immunostaining revealed that epithelial-like CTCs express EpCAM at 8 weeks. Cells were stained with Hoechst 33342, blue for nuclear visualisation. Imaging was performed using an inverted microscope (10×).

A mixture of CTCs (singlets and clusters, Fig. 4a,b respectively) were isolated from the breast cancer patient and cultured in 100 µM CoCl_2_. These cells were visualised using an inverted microscope and seven CTC singlets and two CTC clusters as defined by 3 cells or more consisting of three cells as previously described^22^ were counted. CTC number increased to 55 cells 4 weeks post isolation and reached 150 cells at 8 weeks (Fig. 7a). We observed that CTC viability was maintained for more than 8 weeks. CTCs were immunofluorescently characterised using EpCAM and HER2. Interestingly, the CTCs were EpCAM positive (Fig. 7b), but HER2 was not expressed in these cells.

**Figure 8:**
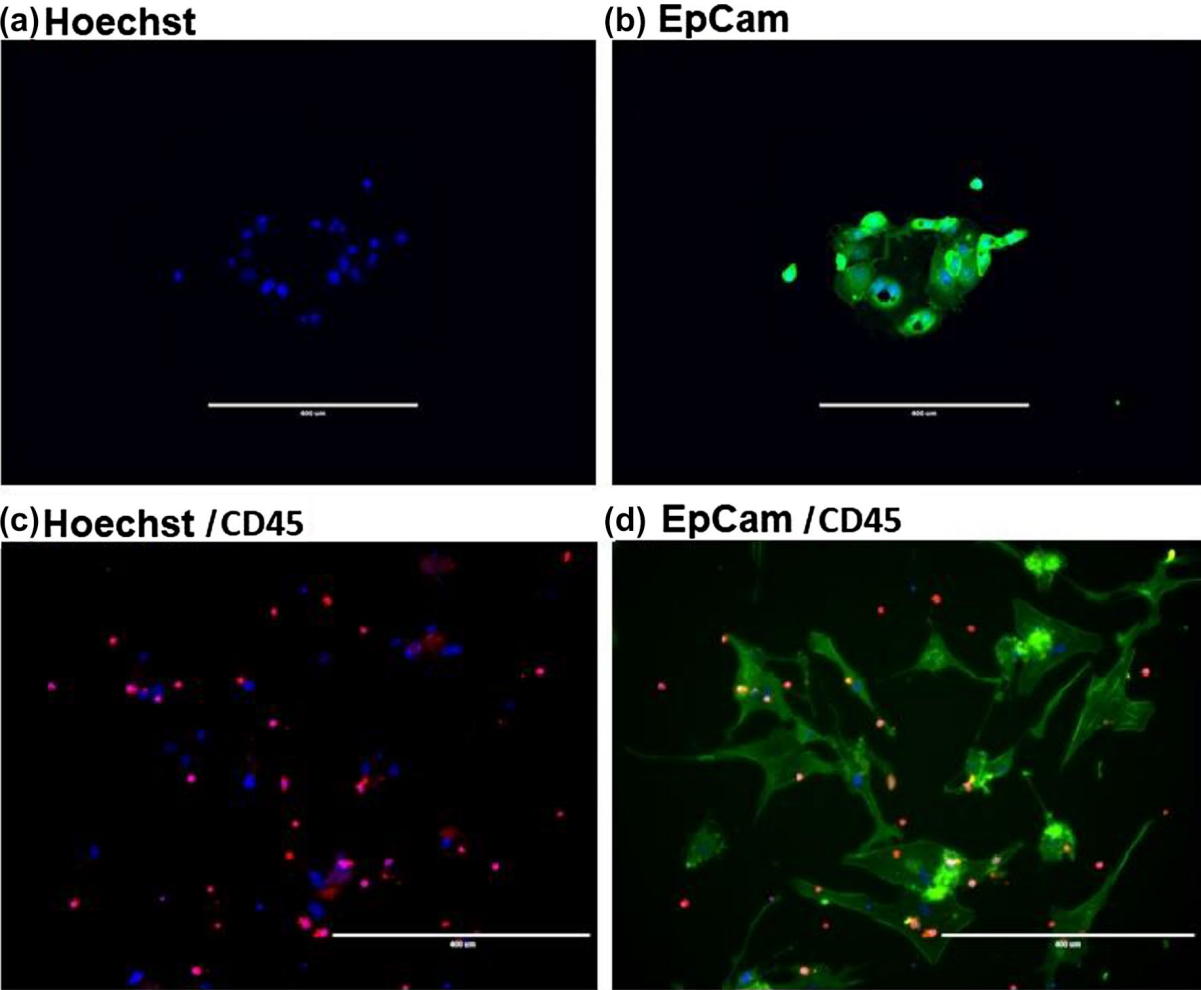
Iimmunofluorescent staining of isolated CTCs captured and isolated from an ovarian cancer patient using the ScreenCell device, (**a**,**b**). CTCs were immunofluorescently stained for (**a**) Hoechst, (**b**) EpCam (green). Ovarian cancer cells isolated from ascites of ovarian cancer patients, (**c**,**d**) . These cells were co-cultured with PBMCs. Following that, cells were fixed in 3% PFA, gently washed with PBS, and probed with anti-EpCam and anti-CD45 antibodies. Our results showed that only cancer cells were EpCam positive (green), while PBMCs were only positively stained with PE-conjugated anti-CD45 antibody (red), and cells were also stained with Hoechst 33342, (blue) for nuclear visualisation. Images were taken with an inverted microscope (10×).

Thus, in the case of the breast cancer patient with quasi-mesenchymal cells in the CTC isolates, we were able to establish long term cultures and demonstrate a very significant expansion of the number of the HER2 positive cells. This provided evidence that the presence of CTC clusters may be critical for the establishment of long-term cultures. Furthermore, we were able to establish ex vivo cultures from low numbers of cells, which is of critical importance due to the low numbers of CTCs in the circulation. For the CTCs isolated from the other six breast cancer patient blood samples, the cells survived in culture for an average of 26.17 days (Range 14 to 35 days). CTCs isolated from ovarian cancer patient blood samples survived for an average of 21 days (Range 7 to 35 days). CTCs survived for 5 weeks from only one ovarian cancer sample. These CTCs were found to be EpCam positive (Fig. 8a,b), with no evidence of CD45 expression. We further confirm these findings by additional examination of the expression of both markers on primary ovarian cancer cells isolated from ascites of ovarian cancer patients. These cells were ex vivo expanded and co-cultured with PBMCs. Then the cells were fixed in 3%PFA and probed with both antibodies (FITC-conjugated anti-EpCam and PE-conjugated anti-CD45 antibodies). We observed that ovarian cancer cells were EpCam positive/CD45 negative whereas PBMCs were CD45 positive/EpCam negative (Fig. 8c,d).

## Discussion

CTCs hold great promise for the early detection of metastatic disease, and to provide a potential precision medicine solution that enables personalised cancer therapy and patient monitoring^7–9^. Despite the potential for CTCs as cutting-edge diagnostic/prognostic/monitoring tools, the clinical utility of CTCs from a liquid biopsy currently comes with significant challenges; which relate to the rarity of CTCs in circulation, issues with CTC isolation/purification, morphological heterogeneity, fragility, and an incomplete knowledge of molecular phenotypes and the effect(s) of homotypic/heterotypic cellular interactions. Several devices for detecting and isolating CTCs lack sensitivity and specificity^9–27^. Long-term culturing and expansion techniques need to be optimised to better understand the morphological and functional properties of CTCs from patient samples, and to enable the development of theranostics.

Since CTCs are thought to originate from a hypoxic environment and can survive in low-adherence conditions, as when circulating in the bloodstream, these two factors may help promote the growth of CTCs in culture systems^27,32^. The key adaptive response to hypoxic conditions is the stabilisation of HIF-1α^45^. HIF-1α protein is overexpressed in solid malignant tumours including breast, colon, gastric, lung, skin, ovarian, pancreatic, prostate, and renal carcinomas when compared to their respective normal tissues^33–37^; consistent with the idea that HIF-1α is upregulated during cancer progression. HIF-1α is a transcription factor that plays a vital role in the functional expression of several genes involved in the adaptation and survival response of cells, tissues, and organs^33–37^. The transition metal cobalt(II) chloride can mimic hypoxia by upregulating and stabilising HIF-1α, which plays an important role in the cell cycle and survival of cancer cells^33–37,45^. HIF is normally regulated by two oxygen‐dependent events that are catalysed by the HIF prolyl 4‐hydroxylases (HIF‐P4Hs). These HIF-P4Hs are 2-oxoglutarate dioxygenases and require Fe^2+^, 2-oxoglutarate, O_2_, and ascorbate for optimal activity^46,47^. CoCl_2_ has been reported to inhibit the activities of HIF-P4Hs, suggesting that it may also occupy this Fe^2+^ binding site and block the degradation of HIF-1α^46,47^. Numerous cellular proteins require iron for activity and in the form of heme and iron-sulfur clusters can act as cofactors for proteins involved in metabolic and regulatory functions. Iron can be transported into cells via a divalent metal transporter and it has been suggested that CoCl_2_ may also be internalised into cells by this same transporter^48^. While intracellular iron levels are not significantly changed following exposure to CoCl_2_^48^, it has been suggested that iron may be required as a catalyst for CoCl_2_ action on HIF-1α. It has been reported that cell culture media supplemented with 10% (v/v) of foetal bovine serum (FCS) contains at least 5 µM of iron^49^.

The expression of HIF-1α was increased when primary ovarian cancer cells were cultured in media supplemented with 20% (v/v) of FCS and exposed to CoCl_2_ at concentrations ranging from 100 to 150 µM. A significant increase in the level of HIF-1α protein at 100 and 150 µM suggested that CoCl_2_ mimics hypoxia in primary ovarian cancer cells by activating HIF-1α expression and/or blocking the degradation of HIF-1α, which normally occurs in other cells in the presence of sufficient oxygen or normoxia^45^. During hypoxia, HIF-1α protein accumulates and binds to hypoxia response elements contained within the promoter region of many genes, such as those that regulate metabolism, cell survival, and angiogenesis^36,37^. While this cell biology supports the growth of cancer in vivo*,* the cell viability and proliferation rate of the primary ovarian cancer cells was also enhanced by exposure to CoCl_2_ at concentrations of 100–150 µM.

Cell cycle analysis showed that the treatment of cells with 200 µM CoCl_2_ induced an accumulation of cells in G0/G1, but this effect was not observed at the lower concentrations. Hypoxia can result in the induction of p21 in many cell types, leading to a G1 arrest^50^. Even though a possible interruption in G2/M has been reported in some hypoxia-stimulated cell lines^51^, our results revealed that the proportion of ovarian cancer cells in G2/M phase was reduced following the exposure to 200 µM of CoCl_2_. Consequently, there was a significant reduction in HIF-1α expression and cell numbers for cultures treated with 200 µM of CoCl_2,_ establishing 100/150 µM CoCl_2_ as the optimal concentration to maintain CTC viability and to effect cell growth.

PHH3 expression was increased at concentrations of 100–150 µM of CoCl_2,_ suggesting that the primary ovarian cancer cells were able to maintain their metabolic and physiological activity under these hypoxia-mimicking conditions. Histone H3 is a nuclear core histone protein of DNA chromatin, with an important role in chromosome condensation and cell-cycle progression during mitosis and meiosis, operating by phosphorylation of serine-10 and serine-28 residues^52,53^. In mammalian cells, in the late G2 phase, phosphorylation begins first in pericentromeric heterochromatin and, as mitosis proceeds, this occurs throughout the entire chromosome; which is completed in the late prophase and maintained through metaphase^44^. There was a significant decrease in PHH3 expression observed with 200 µM CoCl_2,_ confirming the delay of ovarian cancer cells entering the G2/M phase or arrest at the G0/1 phase. CoCl_2_ treatment therefore effectively mimics hypoxia by promoting HIF-1α expression/stability and establishes an environment that activates other downstream molecular machinery involved in cell division.

Hypoxia increases the invasiveness of tumour cells via epithelial-mesenchymal transition^36,37^, which can be monitored by biomarker expression. HIF-1α can enhance the expression of many cancer stem cell phenotype markers as well as the epithelial cell adhesion molecule EpCAM, which can have increased expression during tumour cell growth and metastasis^54,55^. The relatively high abundance of EpCAM expression in CTCs/epithelial cells allows them to be differentiated from white blood cells^56,57^. EpCAM expression was increased under hypoxia conditions for hepatocellular carcinoma and was associated with more aggressive cancer phenotypes, that have altered expression of stem cell markers^55^. However, in our study, the level of expression of EpCAM in untreated/ CoCl_2_ treated primary ovarian cancer cells, as well as in the CTC cultures did not increase, suggesting that a stem cell-like phenotype is being maintained by the hypoxia mimicking culture system.

HER2 is a growth factor receptor which is found to be highly expressed in cancers, particularly those associated with more aggressive disease^58^, and in this study had increased expression in primary ovarian cancer cells treated with CoCl_2_. Zhang et al*.* reported that HER2 was closely related to the proliferation of ovarian cancer cells under conditions of hypoxia^59^. A significant correlation has been reported between HIF-1α and HER2 expression in breast cancers^60,61^, and this was also observed in the CoCl_2_ treated primary ovarian and CTC cultured cells. Activated HER2 phosphorylates many downstream molecules, which in turn activate signalling cascades, including the phosphatidylinositol-3 kinase (PI-3K)/Akt pathway^61^. PI-3K and Akt play an important role in cell survival, tumour growth and progression^62–64^ and also activates the HIF pathway in various tumours^64–66^; playing an important role in proliferation, angiogenesis and metastasis in various types of tumours^36,37^. We established that 100–150 µM CoCl_2_ could be used to stimulate HIF-1α and growth factor receptors such as HER2 and the adhesion molecule EpCAM in order to grow and maintain CTCs isolated from the blood of cancer patients.

CTCs have been successfully isolated from patient’s blood using technologies such as ClearCell, ScreenCell, and others^9,10,13,22^, but the challenge has remained to expand these cells in vitro. For example, CTCs that were isolated using an enhanced mixing chip have only been able to achieve short-term cultures^67^. The method described here overcomes a major obstacle in the field of CTC biology enabling long-term cultures and the expansion of cell numbers and warrants larger studies. This demonstrates that the size-based isolation method can be used to isolate rare CTCs, which can then be successfully cultured; while previous studies have only succeeded in culturing high cell numbers of CTCs^26,27,32^. Furthermore, we used 100 µM of CoCl_2_ to grow two molecular subtypes of breast cancer CTCs; the classical epithelial-like CTCs and the quasi-mesenchymal subtype (also known as the basal-like or squamous subtype). Quasi-mesenchymal CTCs are associated with the poorest prognosis^68,69^ and the relatively higher expression of mesenchymal genes in the quasi-mesenchymal subtype likely contributes to a higher occurrence of epithelial-mesenchymal transition and thus the ability to metastasise, leading to poor outcomes^68,69^. We expanded the number of quasi-mesenchymal CTCs to > 2000 in 8 weeks, demonstrating that the cells were viable and actively proliferating. These cells expressed HER2 but had limited EpCAM expression, which is not surprising, as they take on an EMT phenotype. This downregulation of EpCAM has been found to be associated with epithelial-mesenchymal transition during the formation of metastases^56^. Gorges et al*.* reported faster gene expression changes in CTCs and EpCAM down-regulation which depended on contact between tumour cells and endothelial surfaces or organ systems in in vivo experiments^70^.

In this study, we observed a mixture of epithelial-like CTCs including singlets and clusters and these CTCs were EpCAM-positive HER2-negative. Using the CellSearch System, Fehm et al. reported that 42% of patients with HER2-positive metastatic breast cancer exclusively had HER2-negative CTCs^71^. Recently, a large study by Jaeger et al. demonstrated that 12% of patients with HER2-postive tumours had HER2-negative CTCs^72^. Currently, HER2 status is determined at the time of initial disease diagnosis by analysing primary tumour tissue. HER2 is found to be amplified in 20–30% of human breast cancers^52^. It has been reported that cell–cell junction components such as EpCAM were upregulated in CTC clusters compared to matched CTC singlets^73^. EpCAM was originally identified as a tumour associated antigen on the basis of its high expression level in the tumours of epithelial origin^73^. It has also been reported that EpCAM promotes the proliferation of tumours^74^. These studies supported our findings that the isolated and successfully expanded CTCs were EpCam positive.

The CoCl_2_ low adherence culture method reported here enables rare CTCs to be cultured and expanded, providing a system to improve developmental research and clinical service provision. CTCs, believed to be responsible for seeding and dissemination of cancer, originate from the primary tumour mass and spread in the peripheral circulation among immune cells and erythrocytes^4^. We demonstrated that the viability of isolated epithelial-like CTCs (singlets and clusters) was maintained for more than 8 weeks and that CTCs can be expanded to large numbers using the new culture system. Using our conditions, we were able to establish cultures from low numbers of isolated CTCs in comparison to previous work which successfully cultured from higher numbers of isolated CTCs^27,30^. Furthermore, we suggest that CTC clusters might maintain higher proliferative activity and be more optimal to achieve long term ex vivo cell growth, which may also be consistent with reports that clusters have higher metastatic potential. We had no success with the expansion of cells in long-term culture from patients with only CTC singlets. The presence of EpCAM-positive epithelial-like CTCs and the morphological interactions of CTC clusters/aggregates likely improves the viability and the proliferation activities of these cells or reflects the presence of CTCs with active proliferative potential. CTC clusters have been reported to have a higher proliferation rate compared to single CTCs when assessed using transcriptome analysis and the proliferation marker Ki-67^6^. Additionally, CTC clusters have a survival advantage in the circulation, since the aggregation protects tumour cells from apoptosis, shear stress, and immune response and can facilitate colonisation^2,6^.

Further optimisation of long-term culture conditions with a focus on single CTCs may involve heterotypic and homotypic aggregation. For example, the addition of platelets to CTCs may add a survival advantage as we have previously shown that platelets induce a pro-survival signature^75^. In addition, using a three-dimensional (3D) environment with our optimised hypoxic conditions may improve long-term culture of CTCs. It is well known that 3D culture preserves essential features present in cancer tumours in vivo; i.e. rapid proliferation at the surface and slow metabolism or necrosis in the centre of spheroids.

## Conclusions

We have developed a workflow that can be used to isolate, characterise and culture rare circulating single cells and clusters from the peripheral blood of cancer patients. The culture and expansion of CTCs was facilitated by the creation of a hypoxic environment using CoCl_2_ and low adherence culture system. We also demonstrated the presence of heterogeneous CTC subtypes; a classical epithelial-like CTC and a quasi-mesenchymal subtype isolated from a breast cancer patient’s blood sample. The use of a size-based isolation method allowed the capture of the various phenotypes and CTC clusters that had a survival advantage over single CTCs in our ex vivo culture model system.

## Material and methods

### Ethics

The research protocol was approved by St. James’s Hospital, and Adelaide and Meath Hospital, Dublin, incorporating the National Children’s Hospital Research Ethics Committee (2012/11/04). The experiments were performed in accordance with the Helsinki declaration and relevant guidelines and regulations. Informed consent was obtained from all subjects and/or their legal guardians. All methods in this study were performed in accordance with the relevant guidelines and regulations.

### Ovarian explant and ascites cell culture and expansion

To mimic the in vivo derivation and environmental conditions of CTCs in circulation : a) primary ovarian cancer cells were derived from ovarian cancer cell ex vivo explants and dispersed into monolayers in T-75 tissue culture flasks (VWR, Ireland). The cancer cells were maintained at 37 °C in humidified air balanced with 5% CO_2_ in RPMI 1640 culture medium (GIBCO, Invitrogen, Ireland); supplemented with 10% (v/v) foetal calf serum (Sigma-Aldrich, Ireland), 2 mM l-glutamine and 100 U/mL penicillin–streptomycin (Invitrogen). b) Ovarian cancer cells were isolated from ascites of a high grade serous ovarian patient. Briefly, up to 500 ml of ascites was used to collect cells. Contaminating red blood cells in the cell pellet of ascites were removed by red blood cells lysis buffer (Thermo Fisher Scientific, Dublin, Ireland). The isolated cells were cultured on low attachment 6 well plates (Sarstedt, Nümbrecht, Germany) in supplemented RPMI growth medium as above. Cells were maintained at 37°C in the presence of 5% CO2. Adherent and nonadherent cells were isolated, (nonadherent cells floated as spheroids in the medium), while adherent cells were attached to low attachment plates. After 5 days, floating spheroids and adherent cells were seeded in a 96 well plate for 24h and then co-cultured with human peripheral blood mononuclear cells (PBMCs) from a healthy donor and screened for the expression of EpCAM (cancer cell marker) and CD45 (PBMC marker). 

### Cobalt(II) chloride-mediated HIF-1α induction

A 1 mM stock solution of cobalt(II) chloride (CoCl_2_; Sigma-Aldrich, Ireland) was prepared in dH_2_O and filter sterilised (0.22 µm filter; VWR International Ltd, Dublin, Ireland). Dilutions of this stock solution were prepared in supplemented RPMI 1640 medium (50, 100, 150 and 200 µM). Primary ovarian cancer cells were plated at a density of 7 × 10^3^ cells per well in 96‐well plates, or 2 × 10^6^ in T-75 flasks and allowed to attach for 24 h at 37 °C/5% CO_2_ under normoxic culture conditions. The culture medium was then replaced with CoCl_2_-supplemented RPMI 1640 medium for 48 h at 37 °C/5% CO_2_.

### Protein extraction

Following exposure to CoCl_2_, cells were lysed in RIPA buffer (Trizma Base 50 mM, sodium chloride 150 mM, EDTA 2 mM, and NP40 0.5% v/v) supplemented with a protease inhibitor cocktail (Sigma‐Aldrich Ireland) and incubated on ice for 30 min. Protein samples were centrifuged at 400×*g* for 10 min, at 4 °C and the supernatant collected. Total protein concentration was quantified using the Bradford protein assay (Bio-Rad Laboratories, Hercules, CA, USA), and protein extracts normalised for protein content to 40 µg per sample for either ELISA assay or immunoblotting.

### Quantification of HIF‐1α protein

HIF‐1α protein was determined on ovarian cancer cell extracts by ELISA (HIF‐1α ELISA kit, ThermoFisher Scientific, Dublin, Ireland) according to the manufacturer’s instructions. Absorbance was measured using a spectrophotometer (BIO‐TEK EL808) at 450 nm with a reference wavelength of 550 nm. All samples and standards were analysed in duplicate (technical replicates) to ensure the reliability of single values, and the amount of HIF‐1α protein was calculated by interpolation through a standard curve.

### Immunoblotting

As described above, cells were cultured in T-75 flasks and treated with CoCl_2_ for 48 h. Cell lysates were extracted in RIPA buffer supplemented with a protease inhibitor cocktail (Sigma-Aldrich, Ireland). The resulting lysates were centrifuged at 16,000×*g* for 15 min at 4 °C and the protein content of the supernatants was determined by a Bradford assay. Cell lysates were boiled in Laemmli buffer (final concentration: Tris–HCl 62.5 mM, pH 6.7, Glycerol 10% v/v, sodium dodecyl sulphate 2% w/v, bromophenol blue 0.002% w/v and 143 mM β-mercaptoethanol) for 5 min. Equal amounts of lysates were resolved by sodium dodecyl sulphate polyacrylamide gel electrophoresis (SDS-PAGE). Following electrophoresis, samples were transferred to a polyvinylidene difluoride (PVDF) membrane and blocked overnight in 5% non-fat powdered milk in 0.1% Tween 20/tris-buffered saline (TBST) at 4 °C. The membranes were then probed with mouse anti-phosphorylated histone H3 (PHH3) (Santa Cruz, Biotechnology, Inc, Germany) in 5% non-fat dry milk in TBST overnight at 4 °C, followed by HRP-conjugated anti-mouse (Santa Cruz, Biotechnology, Inc, Germany) for 1 h. The loading control was the constitutively expressed β-actin protein (Santa Cruz, Biotechnology, Inc, Germany). The blots were washed with TBST for 5 min three times followed by one wash with TBS and were visualised using the enhanced chemiluminescence (ECL) system (Thermo Scientific, Dublin, Ireland). The protein bands were visualised using the Fusion FX imaging system (Vilber Lourmat). Densitometric analysis was undertaken using Bio1D software (Vilber Lourmat) with the image analyst blinded with respect to the group designation. Membranes were stripped (62.5 mM, pH 6.8 Tris–HCl, 2% SDS, and 0.83 μl β‐ mercaptoethanol/100 ml) and re‐probed with anti‐β‐actin antibody conjugated to HRP. An EZ‐RUN pre‐stained molecular weight ladder (Fisher Scientific, Dublin, Ireland) was used for molecular weight determination.

### Immunofluorescence staining

Primary ovarian cancer cells were cultured in 96 well plates for 24 h, then the cells were treated with CoCl_2_ (50, 100, 150 and 200 µM) for 48 h. Post-treatment, cells were washed in PBS then fixed in 3% (v/v) PFA and blocked using 3% (w/v) BSA. Cancer cells were then incubated with a FITC-conjugated rabbit anti-human EpCAM (BD BioSciences, US) antibody (1:100), mouse anti-human HER2 (Santa Cruz Biotechnology, Inc, Germany) antibody (1:200) overnight at 4 °C and/or anti-phosphorylated histone H3 (Santa Cruz, Biotechnology, Inc, Germany) (PHH3), anti-vimentin antibody (1:400) and anti-gamma H2AX (γ-H2AX) (S139) antibody (Cell Signaling Technology, Danvers, MA, USA), (1:100) for 1 h at room temperature. Cells were then incubated with either with FITC labelled goat anti-mouse (1:300) or with goat anti-rabbit (1:400) for 1 h at room temperature (Cell Signaling Technology, Danvers, MA, USA respectively). Cells were counterstained with Hoechst 33342 for visualisation of cell nuclei (HQ; 1:1000 dilution; ThermoFisher Scientific, Dublin, Ireland) by incubation for 20 min at room temperature.

### High content screening analysis

The technology is based around automated fluorescence microscopy in combination with advanced image processing and analysis tools^76,77^, which together can provide quantitative information as a first-level description of complex cellular events. To quantify cell viability and the changes in expression of EpCAM and HER2, cells were scanned using a Cytell imaging system (GE Healthcare, UK) and INCell Investigator software (GE Healthcare, UK), respectively.

### Patient samples and blood collection

Blood samples were obtained from 31 treatment-naive patients; 18 breast cancer patients and 13 ovarian cancer patients (Supplementary Tables 1 and 2) at St. James’s Hospital, Dublin 8, Ireland between 2018 and 2020. Breast cancer patients with a preoperative indication at multidisciplinary team discussion for neoadjuvant chemotherapy followed by surgery were recruited and ovarian cancer patients scheduled for either primary debulking surgery or neoadjuvant chemotherapy followed by surgery were recruited. A 6 mL K_2_-EDTA blood sample was taken from each patient for ex vivo culture prior to the initiation of treatment.

### CTC isolation

The ScreenCell LCD kit (ScreenCell, Sarcelles, France) was used to isolate CTCs for long-term culture. In brief, 6 mL of blood was collected from the peripheral vein of patients in K_2_ EDTA-containing tubes. The blood sample was incubated with 1 mL of ScreenCell red blood lysis buffer for 2 min. 1.5 mL of complete culture medium (see “CTC culture” section below) was added to the sample and mixed by inverting the tube once. Samples were then filtered through the ScreenCell Cyto R device as per the manufacturer’s protocol. CTCs were then harvested from the ScreenCell filter by cytospinning them into a 5 mL Eppendorf tube. Samples of isolated cells were resuspended in cultured medium (see “CTC culture” section below) in a 24 well plate, then CTCs were imaged and examined/counted by two independent histopathologists.

### CTC culture

CTCs were cultured in RPMI medium supplemented with 20% foetal bovine serum, 10 µg Insulin, 20 mM HEPES, 10 µM Nicotinamide, 10 μM SB202190, 1.25 mM N-Acetyl-l-cysteine (Sigma-Aldrich, Ireland),10% (v/v) Noggin, 10 ng/mL FGF-10, 1 ng/mL FGF-2, 1X B27 Additive, 1:100 (v/v) Primocin (ThermoFisher Scientific, Dublin, Ireland), and 10 μM Y-27632 (STEMCELL Technology, Vancouver, Canada) and 100 µM CoCl_2_ (Sigma-Aldrich, Ireland) using a 24 well low adherence plate (Sarstedt, Nümbrecht, Germany).

### CTC characterisation post ex vivo culture

Cultured CTCs were washed with PBS and fixed in 3% PFA then blocked using 3% BSA and probed with FITC conjugated rabbit anti-human EpCAM antibody (1:100), unconjugated mouse anti-human HER2 antibody (1:200) and also probed with PE-conjugated anti-CD45 antibody (1:400) using the immunofluorescence method described above.

### Statistical analysis

Statistical analysis was performed with the GraphPad Prism Software v.9.0 (GraphPad, La Jolla, CA, USA). One-way ANOVA followed by Tukey’s post-test and 2-way ANOVA with Bonferroni post-test analysis was carried out on the experimental data, with respect to corresponding controls. A value of *p* < 0.05 was considered statistically significant.

## Supplementary Information

Supplementary Information.

## Data Availability

All data generated or analysed during this study are included in this article (and its supplementary information files).
