# Supplementary material for: Ex vivo expansion of circulating tumour cells (CTCs)
Source: Sci Rep. 2023 Mar 6;13:3704. doi: 10.1038/s41598-023-30733-6 (PMC9988863; doi:10.1038/s41598-023-30733-6)
Supplement: Supplementary file 1 — Supplementary Information. [file 41598_2023_30733_MOESM1_ESM.doc]

**SUPPLEMENTARY INFORMATION**

***Ex Vivo* Expansion of Circulating Tumour Cells (CTCs)**

Bashir M. Mohamed^1,2,3*^, Mark P. Ward^1,2^, Mark Bates^1,2^, Cathy D. Spillane^1,2^, Tanya Kelly^1,2^, Cara Martin^1,2^, Michael Gallagher^1,2^, Sheena Heffernan^1,2^, Lucy Norris^3^, John Kennedy^4^, Feras Abu Saadeh^5^, Noreen Gleeson^5^, Doug A. Brooks^1,2,6^, Robert D. Brooks^6^, Stavros Selemidis^7^, Sharon O’Toole^1,2,3^, John J. O’Leary^1,2^

**AFFILIATIONS**

^1^Department of Histopathology, Trinity College Dublin, Emer Casey Molecular Pathology Research Laboratory, Coombe Women & Infants University Hospital, Dublin, Ireland

^2^ Trinity St James's Cancer Institute, Dublin 8, Ireland

^3^ Department of Obstetrics and Gynaecology, Trinity College Dublin, Dublin, Ireland

^4^HOPE Directorate, St. James’s Hospital, Dublin 8, Ireland

^5^Division of Gynaecological oncology, St. James’s Hospital; Dublin 8, Ireland

^6^Clinical and Health Sciences, University of South Australia, Adelaide, SA 5001, Australia.

^7^School of Health and Biomedical Sciences, RMIT University, Bundoora, Victoria, Australia, 3083.

* Corresponding author: bashmohamed@gmail.com

**Keywords**

Circulating Tumour Cells, hypoxia-inducible factor 1 alpha, Cobalt(II) chloride, long-term culture


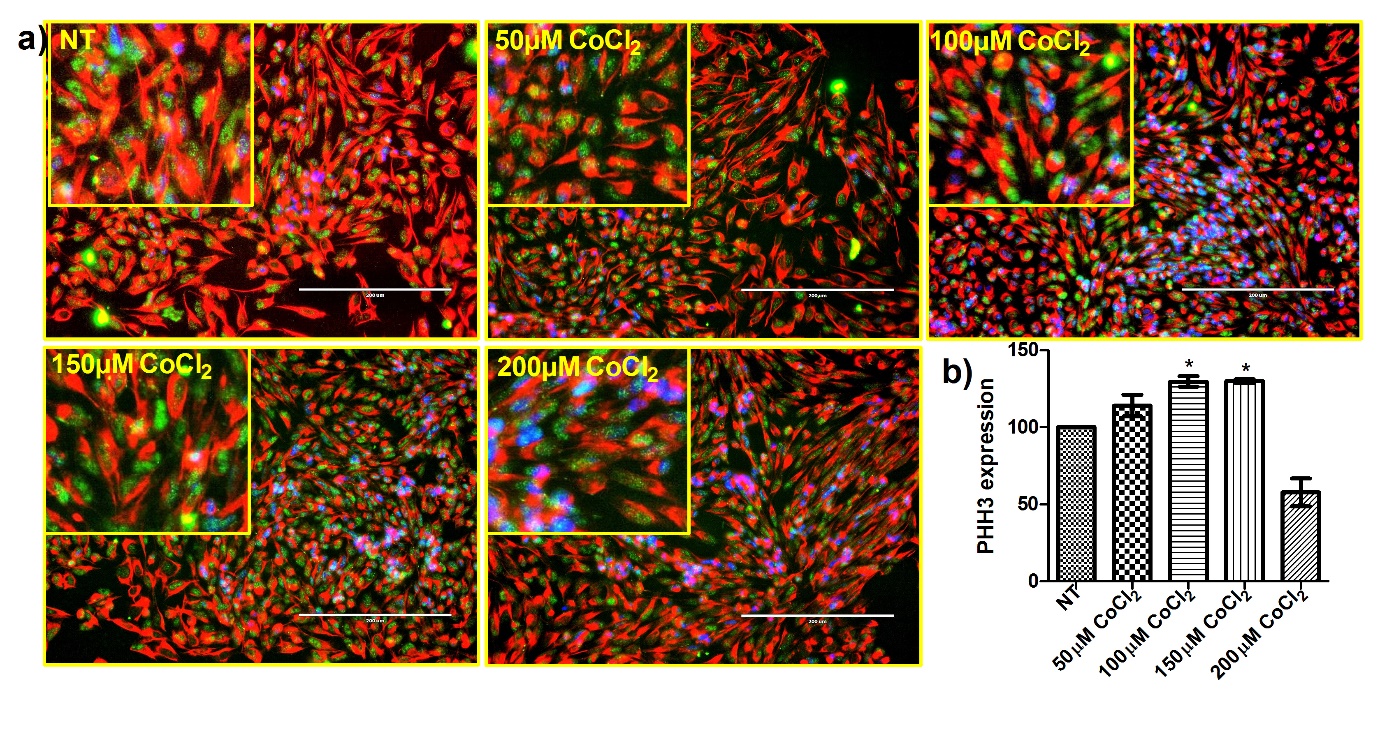
**Supplementary Figure 1.** **Phosphohistone-H3 (PHH3) immunofluorescence**. Primary ovarian cancer cells were incubated with different concentrations of CoCl_2_ (50,100, 150 and 200µM) for 48h. a) Cells were stained with Hoechst 33342 (blue), anti-PHH3 (green) and vimentin (red) and then five microscopic fields per well scanned and analysed using the Cytell imaging system. b) Number of PHH3 stained cells were automatically counted and data were presented as mean ± SEM (n = 3) and were analysed using one‐way ANOVA with Tukey’s post-test relative to corresponding not treated controls (NT), **p* < 0.05.

**
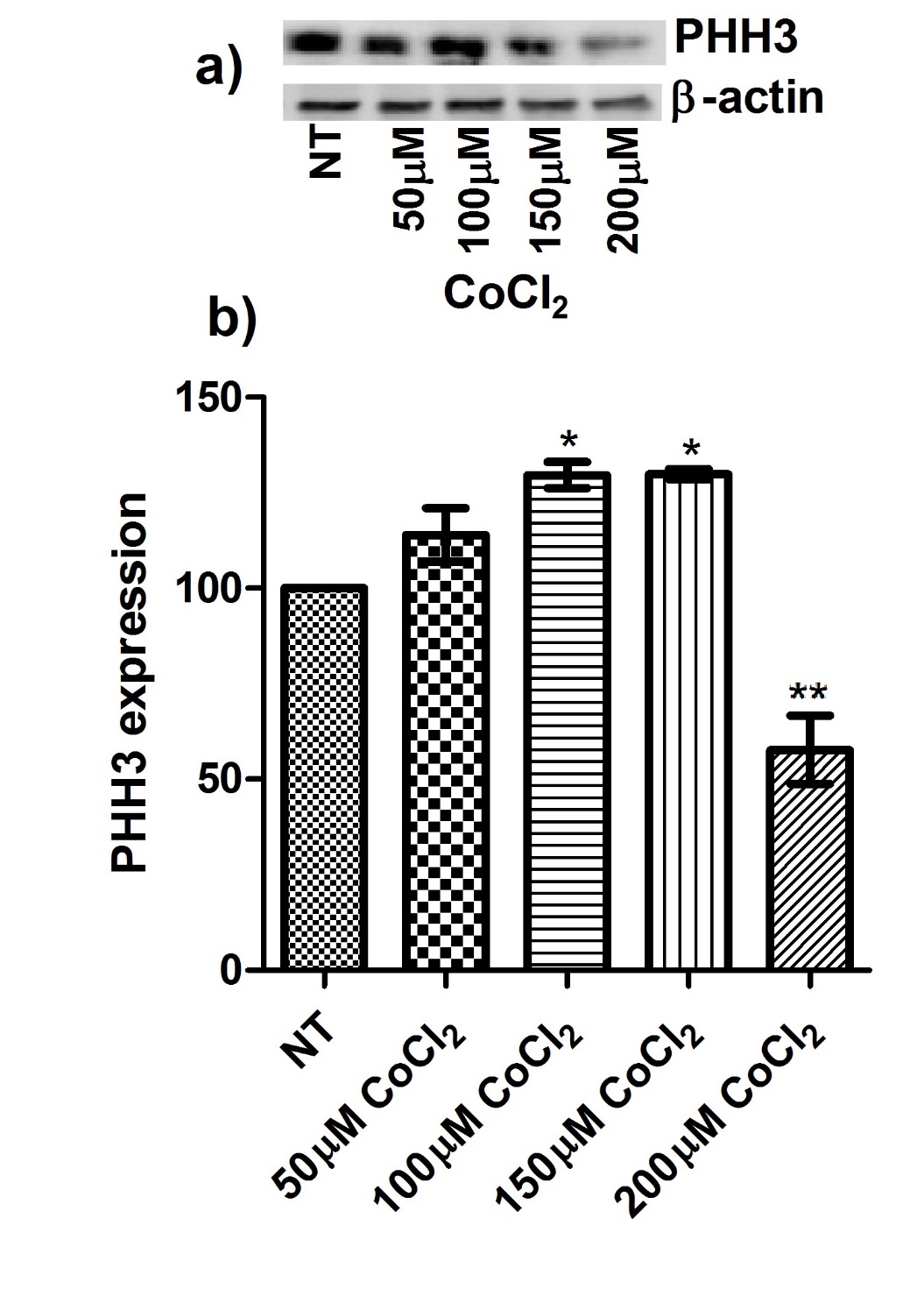
**

**Supplementary Figure 2a.** **Effect of CoCl_2_ on PHH3 expression.** Primary ovarian cancer cells were exposed to different concentrations of CoCl_2_ (50,100,150 and 200 µM) for 48h. a) Cell lysates (40 μg) were resolved by SDS-PAGE and probed with anti-PHH3. b) Relative densitometric analysis of the individual bands was performed. Data were presented as mean ± SEM (n = 3) and were analysed using one‐way ANOVA with Tukey’s post-test carried out on the experimental data, with respect to the corresponding not treated controls (NT), “*” for *p* < 0.05, and “**” for *p* < 0.01.

Rep1

Rep1

**Supplementary Figure 2b**. Raw images of the western blot analysis examining the effect of CoCl_2_ on PHH3 expression.

**Supplementary Table 1.** **Demographic and clinicopathological parameters of patients with breast cancer.**

| Number | Treatment point | Age | Subtype | Grade | Receptor status | Stage |
| --- | --- | --- | --- | --- | --- | --- |
| CTCBR2 | Preneoadjuvant | 45 | Ductal | 2 | ER-, PR-, HER2+ | Locally advanced |
| CTCBR3 | Preneoadjuvant | 47 | Ductal | 1 | ER+, PR+, HER2- | Locally advanced |
| CTCBR4 | Preneoadjuvant | 47 | LOBULAR | 2 | ER+, PR+, HER2- | Locally advanced |
| CTCBR7 | Preneoadjuvant | 57 | Ductal | 2 | ER-, PR-, HER2+ | Locally advanced |
| CTCBR8 | Preneoadjuvant | 52 | Ductal | 3 | ER+, PR+, HER2+ | Locally advanced |
| CTCBR11 | Preneoadjuvant | 39 | Ductal | 3 | ER-, PR-, HER2- | Locally advanced |
| CTCBR12 | Preneoadjuvant | 64 | Ductal | 3 | ER+, PR+, HER2- | Locally advanced |
| CTCBR13 | Preneoadjuvant | 58 | Ductal | 2 | ER+, PR+, HER2- | Locally advanced |
| CTCBR14 | Preneoadjuvant | 30 | Ductal | 3 | ER+, PR+, HER2- | Locally advanced |
| CTCBR17 | Preneoadjuvant | 56 | Ductal | 2 | ER+, PR+, HER2- | Locally advanced |
| CTCBR18 | Preneoadjuvant | 84 | Ductal | 2 | ER+, PR+, HER2+ | Locally advanced |
| CTCBR19 | Preneoadjuvant | 45 | Ductal | 2 | ER+, PR+, HER2- | Locally advanced |
| CTCBR21 | Preneoadjuvant | 51 | Ductal | 3 | ER+, PR-, HER2- | Locally advanced |
| CTCBR22 | Preneoadjuvant | 38 | Ductal | 3 | ER-, PR-, HER2- | Locally advanced |
| CTCBR23 | Preneoadjuvant | 71 | Mixed | 2 | ER+, PR+, HER2+ | Locally advanced |
| CTCBR24 | Preneoadjuvant | 47 | Ductal | 2 | ER-, PR-, HER2- | Locally advanced |
| CTCBR26 | Preneoadjuvant | 55 | Ductal | 2 | ER+, PR+, HER2+ | Locally advanced |
| CTCBR27 | Preneoadjuvant | 52 | Ductal | 3 | ER-, PR-, HER2- | Locally advanced |

**Supplementary Table 2. Demographic and clinicopathological parameters of patients with ovarian cancer.**

| Number | Treatment point | Age | Subtype | Grade | Stage |
| --- | --- | --- | --- | --- | --- |
| CTCOV1 | Preneoadjuvant | 81 | Serous | 3 | 4 |
| CTCOV2 | Pre primary surgery | 42 | Serous | 1 | 1C |
| CTCOV3 | Pre primary surgery | 67 | Serous | 3 | 3C |
| CTCOV4 | Preneoadjuvant | 42 | Serous | 3 | 4B |
| CTCOV5 | Preneoadjuvant | 70 | Serous | 3 | 3C |
| CTCOV6 | Preneoadjuvant | 57 | Serous | 3 | 4 |
| CTCOV11 | Pre primary surgery | 67 | Serous | 3 | 3C |
| CTCOV12 | Preneoadjuvant | 67 | Serous | 3 | 3C |
| CTCOV13 | Pre primary surgery | 57 | Clear | 3 | 4B |
| CTCOV14 | Pre primary surgery | 54 | Serous | 3 | 3C |
| CTCOV15 | Pre primary surgery | 68 | Mixed | 3 | 3C |
| CTCOV17 | Pre primary surgery | 68 | Mixed | 3 | 3B |
| CTCOC18 | Pre primary surgery | 70 | Mixed | 3 | 3C |
